# Gut Microbiota–Informed Precision Nutrition in the Generally Healthy Individual: Are We There Yet?

**DOI:** 10.1093/cdn/nzab107

**Published:** 2021-08-09

**Authors:** Bartek Nogal, Jeffrey B Blumberg, Gil Blander, Milena Jorge

**Affiliations:** InsideTracker, Cambridge, MA, USA; Friedman School of Nutrition Science and Policy, Tufts University, Boston, MA, USA; InsideTracker, Cambridge, MA, USA; InsideTracker, Cambridge, MA, USA

**Keywords:** microbiome, precision nutrition, blood biomarkers, nutrigenomics, generally healthy

## Abstract

Since next generation sequencing facilitated high-throughput and cost-efficient genomics analyses, the human gut metagenome has become an emerging frontier to explore toward precision nutrition. Significant progress has been made in identifying gut microbial features associated with a wide spectrum of human disease. However, other than a few microbiome-disease relations, there is a dearth of confirmed causal inferences, particularly in generally healthy populations. The relatively high unexplained variability in microbiome compositions in this group warrants caution in applying this complex biomarker toward precision nutrition, because our understanding of what constitutes a healthy microbiome is still rudimentary. Although gut microbiota harbor integrated environmental and host-specific information with the potential to facilitate personalized nutritional and lifestyle advice, these data cannot yet be confidently interpreted toward precise recommendations. Thus, nutritional advice for generally healthy individuals based on personal microbiome composition analysis might not yet be appropriate unless accompanied by established blood and physiological biomarkers.

## Teaser

Gut microbiome composition analysis can offer deep layers of health data, but these insights are not yet useful toward precision nutrition for generally healthy individuals.

## Introduction

No longer in its infancy, gut microbiome research is producing insightful results ripe for evaluation toward application to precision nutrition. However, the factors that contribute to a healthy (or unhealthy) state of the intestinal microbiota are dauntingly complex: an interplay of genetic, environmental, clinical, and stochastic inputs can result in 2 seemingly healthy individuals' microbiomes having almost nothing in common from a taxonomic standpoint (1). Although we now have at our disposal a plethora of microbiome-disease association data, there is a need to establish cause-and-effect relations via large-scale longitudinal studies where the initial healthy human microbiome state is functionally characterized and subsequently challenged with interventions whose effects on host microbiota and physiology can then be interrogated for molecular mechanisms. While evaluating whether our current understanding of the microbiome affords us the ability to make microbiome composition–based dietary and lifestyle recommendations, it is worthwhile considering some of the confounding factors that play a role in the variation of the microbiome between individuals. These confounders can significantly impact the utility of microbiome characterization toward iterative health optimization at the level of the generally healthy individual.

**FIGURE 1:**
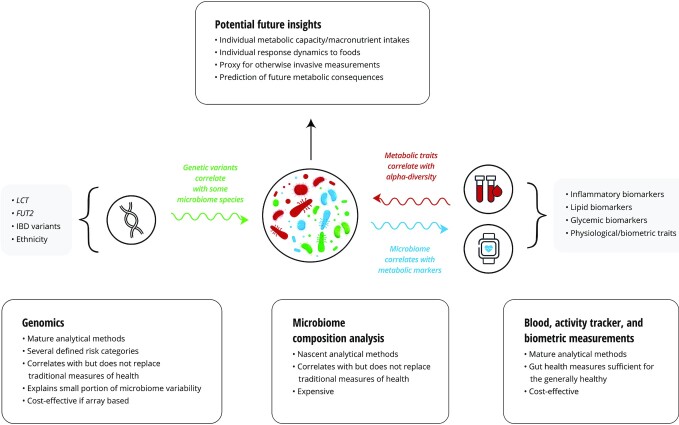
Microbiome composition analysis in generally healthy individuals. In the generally healthy individual, microbiome composition analysis alone currently offers limited actionable health insight that cannot be readily obtained via more traditional means such as blood chemistry and lipids, activity trackers, and basic biometrics. These validated metrics of physiological and metabolic health allow for optimization via established reference ranges that correlate with healthy states, whereas individual-level optimal microbiome features have yet to be elucidated. Often considered to be a marker of health, high α-diversity within the gut microbiome can be qualitatively approximated via traditional measures. However, as this field of research progresses toward the ability to establish optimal microbiome composition baselines at the individual level, metagenomic analysis holds dramatic potential for the practice of precision nutrition. *FUT2*,fucosyltransferase 2; IBD, irritable bowel disease;*LCT*, lactase.

Although this field has progressed exponentially toward more granular datasets, replication of bacterial species-level associations with host phenotypes is scarce, with the most promising reports correlating these associations with easily measured blood biomarkers or phenotypes. Thus, current gut microbial signatures could be just another, albeit highly elaborate, proxy for causal mechanisms behind health states yet to be discovered (**Figure 1**). Because each individual's microbiome is unique, it follows that no 2 persons process the same foods identically and/or derive the same benefit, ultimately making microbiome analysis a possible ideal embodiment of approaches to precision nutrition. However, based on publicly available human microbiome research, we are far from understanding this complex biomarker and thus it is premature for use as an independent nutrition personalization tool.

## Contributors to Microbiome Variation

The term “dysbiosis” is used liberally in the context of microbiome-pathology associations. However, aside from its simplistic definition as a “microbial imbalance,” little is known about the functional aspects of what constitutes an imbalance in the gut microbiome at the level of an individual. Because essentially all references to dysbiosis stem from taxonomic interpretations, there is little mechanistic understanding (2). Before we can confidently label an individual's gut microbiota as in dysbiosis, we need to understand the definition of “eubiosis,” yet another ill-defined (and highly individualized) state corresponding to a well-balanced gut ecosystem. Characterizing an individual's eubiotic state is essential to precision nutrition approaches, particularly in a generally healthy cohort. However, most reports to date have not identified a microbiome core within a population, where individual microbiome diversities tend to fit on a continuum rather than clustering into discrete groups (3). Multiple factors contribute to this variation, and although the scope of this perspective is not inclusive of all the possible contributing personal features to the variability in microbiome compositions, we consider those that might play significant roles.

## Host Genetics and Geographical Location

Individual host genetics contribute a relatively minor portion of the variation within a person's microbiome, with lifestyle factors such as long-term diet substantially outweighing the contribution of any single nucleotide polymorphism (SNP) (4). Xu et al. (5) calculated the heritability of α-diversity to be in a modest range of 3.5–10.3%; when they performed a genome-wide association study (GWAS) for enterotypes, no statistically significant signals were found. These results reflect a consensus that genetics’ impact on a person's microbiome composition is outweighed by lifestyle, geographical, and/or cultural factors (6). However, this does not mean that host genetic variation is not a factor to consider when analyzing generally healthy individuals' microbiomes on the species level. For example, borrowing from the literature of microbiome-disease correlations where the data are plentiful, Mendelian randomization (MR) studies show that genetic predispositions to certain conditions such as chronic kidney disease have causal effects on specific bacterial species abundance (5). Such findings demonstrate the potential of leveraging large GWAS datasets such as the UK Biobank toward understanding similar host genome-microbiota dynamics in the generally healthy population. For example, the results of some MR efforts indicate a significant impact of gut microbiome species on healthspan-related blood phenotypes such as lymphocyte count, eosinophil count, apoA-I, HDL, total cholesterol, BMI, resting heart rate, and blood pressure (7–9). On the other hand, an established SNP in the *LCT* (lactase) gene associated with lactase persistence positively correlates with *Bifidobacterium* abundance, including a large meta-analysis of >18,000 individuals from diverse populations (10). This particular host-microbiome dynamic appears to be a symbiotic compensatory mechanism to facilitate lactose digestion in those who do not produce sufficiently active lactase enzyme (11). Kurilshikov et al. (10) also reported a persistent SNP-microbiome association between *FUT2* (fucosylated mucus glycan secretor/non-secretor) variant and *Ruminococcus torques* genus group. Thus, certain host genetic variations that correlate with the enrichment of particular gut bacterial species can explain host health state phenotypes (such as HDL or BMI); conversely, different subsets of SNPs that associate with particular host predispositions (such as lactose intolerance) can impact the composition of the microbiome. Exploration of such dynamics is in its infancy in generally healthy people and, though the effect sizes are likely to be small relative to the aforementioned nongenomic factors, they could ultimately add more granular insight at the level of individualized interventions. Such detailed analysis could only be useful when the more salient microbiome influencing factors, such as geographical location, are first accounted for.

After examining hundreds of health status phenotypes in >7000 individuals from 14 regions within the Guangdong province of southern China, He et al. (12) found that host location explained the bulk of the microbiota variation. Geographical location was found to explain ∼5 times the variation in the microbiomes relative to the next largest factor, occupation (12). This finding suggests that, in addition to the already challenging task of identifying dysbiosis on the individual level, defining a baseline eubiotic state for an individual would have to account for the region where they live. Thus far, there appears to have been no such effort in the generally healthy populations. However, an approximation of healthy reference ranges within a subset of bacterial taxa yielded CIs that spanned orders of magnitude (13), perhaps in part because no adjustment for geographical location was possible. Although likely still relevant when diagnosing clinical conditions, such wide reference ranges might not yet enable precision approaches to lifestyle interventions until adjusted for geographical region and some of the other confounding factors discussed below.

## Effect of Medication

Reports reveal that there is likely a measurable impact of prescription drugs, over-the-counter medications, and dietary supplements on species-level microbiota, yet the effects of most of these products remain unexplored. Vila et al. (14) reported >150 associations between individual taxa and 17 categories of drugs, with proton pump inhibitors, laxatives, metformin, and vitamin D supplements showing the most associations. Whereas some medications can impact the microbiota directly, for example, as demonstrated for metformin (15), others (such as laxatives) can do so by modifying transit time, which has been cited as one of the strongest explanatory factors for microbiome composition (16). The in vivo picture can be quite dynamic because individuals can modify their behavior based on medication intake; for example, an individual might alter their diet due to nausea or other untoward side effects of the medication or having lower quality of sleep. Thus, precision nutrition approaches to optimize an individual's microbiome should account for intake of medications and dietary supplements at baseline, because shifts in bacterial species induced by these agents could preclude accurate precision nutrition recommendations if they are based on reference populations following different regimens.

## Long-Term Diet Strongly Influences Microbiome Composition

As described in several comprehensive reviews, a plethora of evidence suggests that among the strongest contributors to interindividual host microbiome variation are long-term dietary habits (17–19). The standard Western diet, low in fiber and high in processed ingredients and saturated fat, has been reported to lead to less diverse gut microbiomes and metabolite outputs that appear detrimental to host health. Dietary-induced microbiota composition shifts such as those resulting from a Western diet intervention can lead to exposure to bacterial components toxic to the host (e.g., endotoxins), and result in gut barrier disruption and metabolic endotoxemia (20). In contrast, certain dietary patterns rich in fiber and polyphenols have been shown to have protective effects against the adverse effects of the standard Western diet, in part mediated by an increased production of SCFAs such as butyrate. Some dietary fibers are fermented by bacterial enzymes into SCFAs, which, in addition to supplying enterocytes with energy, act as metabolic signaling molecules and histone deacetylase inhibitors, resulting in immune system modulation, as well as influencing transcription and regulation of appetite (18, 19). Additionally, consumption of protein sources rich in l-carnitine and/or choline leads to the production of trimethylamine (TMA) by the microbiome, which, when oxidized to trimethylamine *N*-oxide in the liver, is associated with an increased risk of cardiovascular disease (18).

## Baseline Microbiome Features and Metabolic Capacity

In the generally healthy population, perhaps the most pertinent application of microbiome analysis is in predicting individual responses to nutritional and lifestyle interventions. If an individual's baseline metabolic capability is known, it might be possible to tailor dietary fiber recommendations, for example, because the same carbohydrates might not benefit all, depending on their baseline microbiome signatures (11). Reports on the effect of probiotic supplementation on the gut microbiome suggest generally beneficial outcomes, although data also suggest a stratification of responders compared with nonresponders to such interventions by baseline microbiome composition and possibly host genetics, as demonstrated with lean donor fecal transplants to individuals with metabolic syndrome (21). Suez et al. (22) further demonstrated that probiotic supplements can delay the re-establishment of a homeostatic gut environment after a course of antibiotics. Our understanding of which microbiome signatures correlate with responsiveness to specific foods is rudimentary. Ultimately, deep knowledge of and multiomics modeling of the metabolic pathways inherent to a particular microbiome signature could allow for tailoring precise dietary interventions that modulate targeted metabolites associated with host health (23). For example, if a TMA-overproducing host microbiome signature is identified, one could minimize sources of carnitine and choline for the individual. On the other hand, another individual might harbor microbes that produce a marked postprandial glucose (PPG) response to starchy carbohydrates while minimizing TMA production. In this case, one might be able to recommend a diet higher in animal protein and lower in starchy carbohydrates.

Specific examples are now available of baseline microbiota-driven dynamics; for example, Korpela et al. (24) reported on particular *Firmicutes* species, *Eubacterium ruminantium* and *Clostridium felsineum*, that correlate with responder compared with nonresponder status. These investigators found that whereas some individuals benefitted from a particular dietary intervention, others showed no or even adverse responses, each explained by their baseline microbiomes (24). Similar findings from the Weitzman Institute in Israel have linked gut microbial signatures to postprandial glucose responses (PPGRs), where microbiome features strongly correlate with blood sugar responses to foods (25). Other results revealed that white bread can be advantageous to some over traditional rye wheat bread based on personalized microbiome signatures (26). This research is beginning to validate the adage that “one man's cure is another man's poison.” Nonetheless, replication of these findings is warranted in larger populations to enable personalized recommendations in diverse groups.

To validate the microbiome-PPG algorithm described above, Mendes-Soares et al. (27) implemented the same methods reported by Zeevi et al. (25) in 327 free-living American Midwesterners followed for PPGRs to foods. Although the data were qualitatively replicated, with the microbiome contribution predicting PPRGs at *r* = 0.62, the model underperformed relative to that in the Israeli population, suggesting that further research is needed before we can confidently use microbiome biomarkers as accurate predictors of PPGRs across populations (28). In an independent cohort of >1000 deeply phenotyped individuals, Asnicar et al. (29) attempted a comprehensive evaluation of the interplay of long-term diet, microbiome composition, and hundreds of fasting and periprandial cardiometabolic blood biomarkers. They only partially replicated the Israeli cohort findings by Zeevi et al. (25), with overall microbiome features explaining relatively little of the variation of glycemic indexes relative to blood lipids and inflammatory biomarkers (25, 29). The PPGRs showed a marginal association with the gut microbiome (AUC = 0.6), again potentially highlighting the importance of examining different populations toward replicating microbiome-phenotype associations. This task is not trivial because host–microbe dynamics are complex and results can seem contradictory depending on the details of what parameters are being measured. For example, in addition to demonstrating significant associations of food groups and habitual diet with microbiome features, Asnicar et al. (29) noted differential effects of certain bacterial species of fasting compared with postprandial rises in biomarkers: *Flavonifractor plautii* was associated with increased systemic inflammation biomarkers such as fasting GlycA [composite marker of systemic inflammation based on an NMR signature of select acute phase proteins in the blood], but this was decoupled from the biomarker's postprandial rise, where the same species was correlated with a decrease in GlycA. Several other immunological and some blood lipid biomarkers followed an analogous microbe–blood metabolite dynamic (29). These results highlight the intricate complexity of the microbiome and the need to better understand mechanisms before using an individual's microbiome composition in the context of precision nutrition.

Certain microbiome features whose association with host health has been relatively consistently replicated in the literature might still not be ready for implementation in precision nutrition platforms. One such example is the commensal *Akkermansia muciniphila*, a well-characterized gut bacterial species that has shown potential for some clinical utility with regard to obesity (30). In the trial conducted by Asnicar et al. (29) encompassing >1000 deeply phenotyped individuals, this beneficial species was not among the main players correlating with cardiometabolic health. However, a proof-of-concept clinical trial using both live and pasteurized versions of an *A. muciniphila* probiotic did show slightly improved insulin sensitivity, reduced insulinemia and total cholesterol, as well as fat mass reduction in individuals with obesity (30). These results demonstrate the potential of microbiome analysis for facilitating novel probiotic approaches toward improving cardiometabolic health outcomes. However, they also highlight our distance from being able to rely on an individual's gut species abundance metrics for improving health outcomes. This lack of replication of species-level microbiome results will need to be addressed before such results can be reliably applied to precision nutrition platforms. This dearth of replication can, in part, be explained by factors that confound microbiome analysis.

## Stochastic Effects and Confounders of Microbiome Composition Analysis

The majority of human microbiome studies fall short of assigning causal effects of the gut microbial environment on host phenotypes (31). Thus, it cannot be discerned whether the gut microbiota composition was affected prior to an intervention or whether the bacterial populations present are a consequence of the intervention itself. This situation is in contrast to studies using rodent models, where investigators often report a transfer of pathological phenotype, but then make often misplaced causal inferences with regards to human hosts (32). Indeed, animal-based microbiome research might have played a role in overstating the causal effects of the microbiota in human health and disease. Moreover, there is a paucity of longitudinal studies tracking shifts in the gut microbial populations toward developing the concept of a core healthy microbiome. David et al. (33) undertook such an exercise in mapping the effects of 10,000 longitudinal human wellness measurements to daily gut and salivary microbiota shifts through a period of 1 y for 2 individuals. They confirmed that, on the community level, the microbiome was stable on a scale of months but noted that activities such as travel throughout various parts of the world can instill profound changes. In addition to the aforementioned strong influence of geographical location on microbiome composition, multiple reports have indicated that the microbiome does entrain on diurnal rhythms, which, if disrupted by interventions such as jet lag or sleep loss, can lead to dysbiosis (34). Caporaso et al. (35) reported a marked variability in 2 individuals’ microbiota at the sequencing depth examined, suggesting that no core microbiome exists at high abundance because only a small subset of the bacterial taxa were found to be consistently present across all samples even day-to-day. Others who have examined larger cohorts (though at reduced sampling frequency) have suggested that a single measurement of the unperturbed fecal microbiome can supply long-term insight on composition and metabolic potential (36). The apparently different conclusions drawn by these researchers could be inherent to the respective study designs: fewer subjects enable more frequent sampling and can reveal day-to-day variance, whereas larger studies are not powered (due to cost) to detect significant longitudinal variances. In practice, the sampling frequency can ultimately be dictated by pragmatism and individual lifestyle dynamics. Importantly, any approach should ensure that samples are processed using identical procedures leading up to point of data analysis.

As noted, the reproducibility of microbiome associations across independent studies is low, with a significant methodological confounder being the DNA extraction step (10). Other confounding variables include improper and/or inconsistent documentation of sample collection and processing, and yet-to-be standardized data processing and analysis methods (1). It appears clear that stool consistency, described with the Bristol Stool Index (BSI), is often referred to as the single personal factor with the largest effect size on microbiome composition variation in healthy individuals (37). Further, some estimates put the cumulative explanatory power of wellness and lifestyle variables on interindividual microbiota variation at <8% (38). This suggests that much of the variation remains unexplained, with some reports proposing that microbiome-host associations reported to date are overstated (39). As part of their suggested remedy to this challenge, Vujkovic-Cvijin et al. (39) have offered an essential methodological approach to data analysis that involves case-control matching for confounding variables that strongly associate with microbiome composition. They point out that much of the difference in microbiomes between disease cases, such as type 2 diabetes and irritable bowel syndrome, and controls is diminished when both are matched for alcohol intake or BSI. Approaching independent datasets with consistent analytical rigor can help reduce spurious findings and increase the number of studies that are able to reproduce associations between health status and gut microbiota, thus beginning to make this complex metagenomic biomarker also ripe for more scalable precision nutrition applications in the generally healthy.

## Concluding Remarks

The determination of individual microbiome composition holds potential to be an important tool for precision nutrition in addition to the currently available personal data derived from blood and various genome, epigenome, metabolome, and emerging glycomic biomarkers of health and healthspan. However, until microbiota associations are more consistently replicated, and randomized clinical trials and/or other longitudinal cohort approaches revealing causal effects of modifying the microbiome on wellness (not just clinical) phenotypes, the interpretation of individual microbiomes toward personalized recommendations remains a challenge. Importantly, because intraindividual microbiome composition variability has been established as much lower than interindividual differences, it follows that each individual should harbor personalized species abundance averages that define their state of health. Nonetheless, even within-person microbiome variability can be partly a function of the frequency of sampling. Thus, temporal variability should be adjusted in longitudinal samples toward establishing individualized bacterial abundance metrics that would allow for more accurate assessments of metabolic capacities and facilitate “thresholds” for triggering personalized recommendations. Until microbiome analysis matures to a stage where consistent bacterial species’ functional effects have been demonstrated in independent studies spanning various demographics, it appears to be premature for metagenomics, in and of itself, to serve as a cost-effective solution or a reliable biomarker of wellness in individuals who are generally healthy. However, casting a wide ‘omics net, including metagenomics at the individual level, in future longitudinal studies, such as the NIH Nutrition for Precision Health program, should enable *n*-of-1 study approaches toward realizing the microbiome's full potential in precision nutrition (40).

## Notes

InsideTracker is the sole funding source.

Author disclosures: BN, GB, and MJ are employees of InsideTracker. JBB serves on the Scientific Advisory Board of InsideTracker.

Abbreviations used: BSI, Bristol Stool Index; GlycA, composite marker of systemic inflammation based on an NMR signature of select acute phase proteins in the blood; GWAS, genome-wide association study; MR, Mendelian randomization; PPG, postprandial glucose; PPGR, postprandial glucose response; SNP, single nucleotide polymorphism; TMA, trimethylamine.

## References

[1] Gilbert JA, Blaser MJ, Caporaso JG, Jansson JK, Lynch SV, Knight R. Current understanding of the human microbiome. Nat Med. 2018;24(4):392–400. 29634682 10.1038/nm.4517 PMC7043356

[2] Weiss GA, Hennet T. Mechanisms and consequences of intestinal dysbiosis. Cell Mol Life Sci. 2017;74(16):2959–77. 28352996 10.1007/s00018-017-2509-x PMC11107543

[3] Jeffery IB, Claesson MJ, O'Toole PW, Shanahan F. Categorization of the gut microbiota: enterotypes or gradients?. Nat Rev Microbiol. 2012;10(9):591–2. 23066529 10.1038/nrmicro2859

[4] Goodrich JK, Davenport ER, Clark AG, Ley RE. The relationship between the human genome and microbiome comes into view. Annu Rev Genet. 2017;51(1):413–33. 28934590 10.1146/annurev-genet-110711-155532 PMC5744868

[5] Xu F, Fu Y, Sun TY, Jiang Z, Miao Z, Shuai M, Gou W, Ling CW, Yang J, Wang J et al. The interplay between host genetics and the gut microbiome reveals common and distinct microbiome features for complex human diseases. Microbiome. 2020;8(1):145. 33032658 10.1186/s40168-020-00923-9 PMC7545574

[6] Gupta VK, Paul S, Dutta C. Geography, ethnicity or subsistence-specific variations in human microbiome composition and diversity. Front Microbiol. 2017;8:1162. 28690602 10.3389/fmicb.2017.01162 PMC5481955

[7] Groot HE, van de Vegte YJ, Verweij N, Lipsic E, Karper JC, van der Harst P. Human genetic determinants of the gut microbiome and their associations with health and disease: a phenome-wide association study. Sci Rep. 2020;10(1):14771. 32901066 10.1038/s41598-020-70724-5 PMC7479141

[8] Li X, Ploner A, Wang Y, Zhan Y, Pedersen NL, Magnusson PK, Jylhava J, Hagg S. Clinical biomarkers and associations with healthspan and lifespan: evidence from observational and genetic data. EBioMedicine. 2021;66:103318. 33813140 10.1016/j.ebiom.2021.103318 PMC8047464

[9] Levine ME, Lu AT, Quach A, Chen BH, Assimes TL, Bandinelli S, Hou L, Baccarelli AA, Stewart JD, Li Y et al. An epigenetic biomarker of aging for lifespan and healthspan. Aging. 2018;10(4):573–91. 29676998 10.18632/aging.101414 PMC5940111

[10] Kurilshikov A, Medina-Gomez C, Bacigalupe R, Radjabzadeh D, Wang J, Demirkan A, Le Roy CI, Raygoza Garay JA, Finnicum CT, Liu X et al. Large-scale association analyses identify host factors influencing human gut microbiome composition. Nat Genet. 2021;53(2):156–65. 33462485 10.1038/s41588-020-00763-1 PMC8515199

[11] Kolodziejczyk AA, Zheng D, Elinav E. Diet-microbiota interactions and personalized nutrition. Nat Rev Microbiol. 2019;17(12):742–53. 31541197 10.1038/s41579-019-0256-8

[12] He Y, Wu W, Zheng HM, Li P, McDonald D, Sheng HF, Chen MX, Chen ZH, Ji GY, Zheng ZD et al. Regional variation limits applications of healthy gut microbiome reference ranges and disease models. Nat Med. 2018 Oct;24(10):1532–5. 30150716 10.1038/s41591-018-0164-x

[13] Almonacid DE, Kraal L, Ossandon FJ, Budovskaya YV, Cardenas JP, Bik EM, Goddard AD, Richman J, Apte ZS. 16S rRNA gene sequencing and healthy reference ranges for 28 clinically relevant microbial taxa from the human gut microbiome. PLoS One. 2017;12(5):e0176555. 28467461 10.1371/journal.pone.0176555 PMC5414997

[14] Vila AV, Collij V, Sanna S, Sinha T, Imhann F, Bourgonje AR, Mujagic Z, Jonkers D, Masclee AAM, Fu J et al. Impact of commonly used drugs on the composition and metabolic function of the gut microbiota. Nat Commun. 2020;11(1):362. 31953381 10.1038/s41467-019-14177-z PMC6969170

[15] Elbere I, Kalnina I, Silamikelis I, Konrade I, Zaharenko L, Sekace K, Radovica-Spalvina I, Fridmanis D, Gudra D, Pirags V et al. Association of metformin administration with gut microbiome dysbiosis in healthy volunteers. PLoS One. 2018;13(9):e0204317. 30261008 10.1371/journal.pone.0204317 PMC6160085

[16] Muller M, Canfora EE, Blaak EE. Gastrointestinal transit time, glucose homeostasis and metabolic health: modulation by dietary fibers. Nutrients. 2018;10(3):275. 10.3390/nu10030275 PMC5872693 29495569

[17] Wilson AS, Koller KR, Ramaboli MC, Nesengani LT, Ocvirk S, Chen C, Flanagan CA, Sapp FR, Merritt ZT, Bhatti F et al. Diet and the human gut microbiome: an international review. Dig Dis Sci. 2020;65(3):723–40. 32060812 10.1007/s10620-020-06112-w PMC7117800

[18] Frame LA, Costa E, Jackson SA. Current explorations of nutrition and the gut microbiome: a comprehensive evaluation of the review literature. Nutr Rev. 2020;78(10):798–812. 32211860 10.1093/nutrit/nuz106

[19] Voreades N, Kozil A, Weir TL. Diet and the development of the human intestinal microbiome. Front Microbiol. 2014;5:494. 25295033 10.3389/fmicb.2014.00494 PMC4170138

[20] Candido TLN, Bressan J, Alfenas RCG. Dysbiosis and metabolic endotoxemia induced by high-fat diet. Nutr Hosp. 2018;35(6):1432–40. 30525859 10.20960/nh.1792

[21] Kootte RS, Levin E, Salojarvi J, Smits LP, Hartstra AV, Udayappan SD, Hermes G, Bouter KE, Koopen AM, Holst JJ et al. Improvement of insulin sensitivity after lean donor feces in metabolic syndrome is driven by baseline intestinal microbiota composition. Cell Metab. 2017;26(4):611–19.e6. 28978426 10.1016/j.cmet.2017.09.008

[22] Suez J, Zmora N, Zilberman-Schapira G, Mor U, Dori-Bachash M, Bashiardes S, Zur M, Regev-Lehavi D, Ben-Zeev Brik R, Federici S et al. Post-antibiotic gut mucosal microbiome reconstitution is impaired by probiotics and improved by autologous FMT. Cell. 2018;174(6):1406–23. 30193113 10.1016/j.cell.2018.08.047

[23] Wilmanski T, Rappaport N, Diener C, Gibbons SM, Price ND. From taxonomy to metabolic output: what factors define gut microbiome health?. Gut Microbes. 2021;13(1):1–20. 10.1080/19490976.2021.1907270 PMC8078686 33890557

[24] Korpela K, Flint HJ, Johnstone AM, Lappi J, Poutanen K, Dewulf E, Delzenne N, de Vos WM, Salonen A. Gut microbiota signatures predict host and microbiota responses to dietary interventions in obese individuals. PLoS One. 2014;9(3):e90702. 24603757 10.1371/journal.pone.0090702 PMC3946202

[25] Zeevi D, Korem T, Zmora N, Israeli D, Rothschild D, Weinberger A, Ben-Yacov O, Lador D, Avnit-Sagi T, Lotan-Pompan M et al. Personalized nutrition by prediction of glycemic responses. Cell. 2015;163(5):1079–94. 26590418 10.1016/j.cell.2015.11.001

[26] Korem T, Zeevi D, Zmora N, Weissbrod O, Bar N, Lotan-Pompan M, Avnit-Sagi T, Kosower N, Malka G, Rein M et al. Bread affects clinical parameters and induces gut microbiome-associated personal glycemic responses. Cell Metab. 2017;25(6):1243–53. 28591632 10.1016/j.cmet.2017.05.002

[27] Mendes-Soares H, Raveh-Sadka T, Azulay S, Ben-Shlomo Y, Cohen Y, Ofek T, Stevens J, Bachrach D, Kashyap P, Segal L et al. Model of personalized postprandial glycemic response to food developed for an Israeli cohort predicts responses in Midwestern American individuals. Am J Clin Nutr. 2019;110(1):63–75. 31095300 10.1093/ajcn/nqz028 PMC6599737

[28] Mendes-Soares H, Raveh-Sadka T, Azulay S, Edens K, Ben-Shlomo Y, Cohen Y, Ofek T, Bachrach D, Stevens J, Colibaseanu D et al. Assessment of a personalized approach to predicting postprandial glycemic responses to food among individuals without diabetes. JAMA Netw Open. 2019;2(2):e188102. 30735238 10.1001/jamanetworkopen.2018.8102 PMC6484621

[29] Asnicar F, Berry SE, Valdes AM, Nguyen LH, Piccinno G, Drew DA, Leeming E, Gibson R, Le Roy C, Khatib HA et al. Microbiome connections with host metabolism and habitual diet from 1,098 deeply phenotyped individuals. Nat Med. 2021;27(2):321–32. 33432175 10.1038/s41591-020-01183-8 PMC8353542

[30] Depommier C, Everard A, Druart C, Plovier H, Van Hul M, Vieira-Silva S, Falony G, Raes J, Maiter D, Delzenne NM et al. Supplementation with *Akkermansia muciniphila* in overweight and obese human volunteers: a proof-of-concept exploratory study. Nat Med. 2019;25(7):1096–103. 31263284 10.1038/s41591-019-0495-2 PMC6699990

[31] Fischbach MA. Microbiome: focus on causation and mechanism. Cell. 2018;174(4):785–90. 30096310 10.1016/j.cell.2018.07.038 PMC6094951

[32] Walter J, Armet AM, Finlay BB, Shanahan F. Establishing or exaggerating causality for the gut microbiome: lessons from human microbiota-associated rodents. Cell. 2020;180(2):221–32. 31978342 10.1016/j.cell.2019.12.025

[33] David LA, Materna AC, Friedman J, Campos-Baptista MI, Blackburn MC, Perrotta A, Erdman SE, Alm EJ. Host lifestyle affects human microbiota on daily timescales. Genome Biol. 2014;15(7):R89. 25146375 10.1186/gb-2014-15-7-r89 PMC4405912

[34] Nobs SP, Tuganbaev T, Elinav E. Microbiome diurnal rhythmicity and its impact on host physiology and disease risk. EMBO Rep. 2019;20:e47129. 30877136 10.15252/embr.201847129 PMC6446202

[35] Caporaso JG, Lauber CL, Costello EK, Berg-Lyons D, Gonzalez A, Stombaugh J, Knights D, Gajer P, Ravel J, Fierer N et al. Moving pictures of the human microbiome. Genome Biol. 2011;12(5):R50. 21624126 10.1186/gb-2011-12-5-r50 PMC3271711

[36] Mehta RS, Abu-Ali GS, Drew DA, Lloyd-Price J, Subramanian A, Lochhead P, Joshi AD, Ivey KL, Khalili H, Brown GT et al. Stability of the human faecal microbiome in a cohort of adult men. Nat Microbiol. 2018;3(3):347–55. 29335554 10.1038/s41564-017-0096-0 PMC6016839

[37] Gilbert JA, Alverdy J. Stool consistency as a major confounding factor affecting microbiota composition: an ignored variable?. Gut. 2016;65(1):1–2. 26187505 10.1136/gutjnl-2015-310043

[38] Falony G, Joossens M, Vieira-Silva S, Wang J, Darzi Y, Faust K, Kurilshikov A, Bonder MJ, Valles-Colomer M, Vandeputte D et al. Population-level analysis of gut microbiome variation. Science. 2016;352(6285):560–4. 27126039 10.1126/science.aad3503

[39] Vujkovic-Cvijin I, Sklar J, Jiang L, Natarajan L, Knight R, Belkaid Y. Host variables confound gut microbiota studies of human disease. Nature. 2020;587(7834):448–54. 33149306 10.1038/s41586-020-2881-9 PMC7677204

[40] National Institutes of Health. Nutrition for Precision Health, powered by the All of Us research program. [Internet]. [cited March 2, 2021]. Available from: https://commonfund.nih.gov/nutritionforprecisionhealth.

